# Comparative Temporal Analysis of Morbidity and Early Mortality in Heart Transplantation with Extracorporeal Membrane Oxygenation Support: Exploring Trends over Time

**DOI:** 10.3390/biomedicines12092109

**Published:** 2024-09-16

**Authors:** Raquel López-Vilella, Manuel Pérez Guillén, Borja Guerrero Cervera, Ricardo Gimeno Costa, Iratxe Zarragoikoetxea Jauregui, Francisca Pérez Esteban, Paula Carmona, Tomás Heredia Cambra, Mónica Talavera Peregrina, Azucena Pajares Moncho, Carlos Domínguez-Massa, Víctor Donoso Trenado, Luis Martínez Dolz, Pilar Argente, Álvaro Castellanos, Juan Martínez León, Salvador Torregrosa Puerta, Luis Almenar Bonet

**Affiliations:** 1 Heart Failure and Transplant Unit, Hospital Universitari i Politècnic La Fe, 46026 Valencia, Spain; vdonoso@outlook.com (V.D.T.); lualmenar@gmail.com (L.A.B.); 2 Cardiology Department, Hospital Universitari i Politècnic La Fe, 46026 Valencia, Spain; borja_vlc95@hotmail.com (B.G.C.); martinez_luidol@gva.es (L.M.D.); 3 Cardiovascular Surgery Department, Hospital Universitari i Politècnic La Fe, 46026 Valencia, Spain; perez_mgui@gva.es (M.P.G.); tomheca@hotmail.com (T.H.C.); dominguez.massa@gmail.com (C.D.-M.); juan.martinez-leon@uv.es (J.M.L.); torregrosa_sal@gva.es (S.T.P.); 4 Intensive Care Department, Hospital Universitari i Politècnic La Fe, 46026 Valencia, Spain; ricardogimeno55@hotmail.com (R.G.C.); perez_fraest@gva.es (F.P.E.); talavera_mon@gva.es (M.T.P.); castellanos_alv@gva.es (Á.C.); 5 Department of Anesthesiology and Critical Care, Hospital Universitari i Politècnic La Fe, 46026 Valencia, Spain; iratxezarragoikoetxea@hotmail.com (I.Z.J.); paulac_g@hotmail.com (P.C.); pajares.maz@gmail.com (A.P.M.); argente_marnav@gva.es (P.A.); 6 Centro de Investigación Biomédica en Red de Enfermedades Cardiovasculares (CIBERCV), Instituto de Salud Carlos III, 28029 Madrid, Spain

**Keywords:** urgent heart transplantation, venoarterial extracorporeal membrane oxygenation, morbidity, mortality, eras

## Abstract

Background/Objectives: The direct bridge to urgent heart transplant (HT) with venoarterial extracorporeal membrane oxygenation (VA-ECMO) support has been associated with high morbidity and mortality. The objective of this study is to analyze the morbidity and mortality of patients transplanted with VA-ECMO and compare the presumed differences between various eras over a 17-year timeline. Methods: This is a prospective, observational study on consecutive patients stabilized with VA-ECMO and transplanted with VA-ECMO from July 2007 to December 2023 at a reference center (98 patients). Objective variables were mortality and morbidity from renal failure, venous thromboembolic disease (VTD), primary graft dysfunction (PGD), the need for tracheostomy, severe myopathy, reoperation, post-transplant ECMO, vascular complications, and sepsis/infection. Results: The percentage of patients who reached transplantation without the need for mechanical ventilation has increased over the periods studied. No significant differences were found between the study periods in 30-day mortality (*p* = 0.822), hospital discharge (*p* = 0.972), one-year mortality (*p* = 0.706), or five-year mortality (*p* = 0.797). Survival rates in these periods were 84%, 75%, 64%, and 61%, respectively. Comorbidities were very frequent, with an average of 3.33 comorbidities per patient. The most frequent were vascular complications (58%), the need for post-transplant ECMO (57%), and myopathy (55%). The development of myopathy and the need for post-transplant ECMO were higher in recent periods (*p* = 0.004 and *p* = 0.0001, respectively). Conclusions: VA-ECMO support as a bridge to HT allows hospital discharge for 3 out of 4 transplanted patients. This survival rate has not changed over the years. The comorbidities associated with this device are frequent and significant.

## 1. Introduction

Cardiogenic shock (CS) has been, for decades, one of the greatest challenges in critical cardiology. This condition presents a very high risk of significant morbidity and mortality despite the most current therapeutic advances [1,2]. In this context, circulatory assistance with extracorporeal membrane oxygenation in venoarterial configuration [VA-ECMO] has undoubtedly been a crucial intervention to provide circulatory and oxygenation support in refractory CS, especially in INTERMACS 1–2 cases [1,3,4]. In cases of non-recovery of myocardial function, an urgent heart transplant (HT) is often performed in patients supported with ECMO [5]. However, the morbidity associated with ECMO assistance is not negligible and is exacerbated by the complex profile of the patients who require its use [6,7,8]. In high-volume centers for VA-ECMO implantation, complications can be minimized, and it is possible that over time and with the team’s experience, the survival and complications of patients undergoing HT with ECMO have changed. The objective of this study is to analyze the early global morbidity, mortality and eras of patients undergoing HT assisted by VA-ECMO in a long-term time series (17 years). The secondary objective is to analyze mid- to long-term mortality by time periods in the same patients.

## 2. Materials and Methods

**Figure 1:**
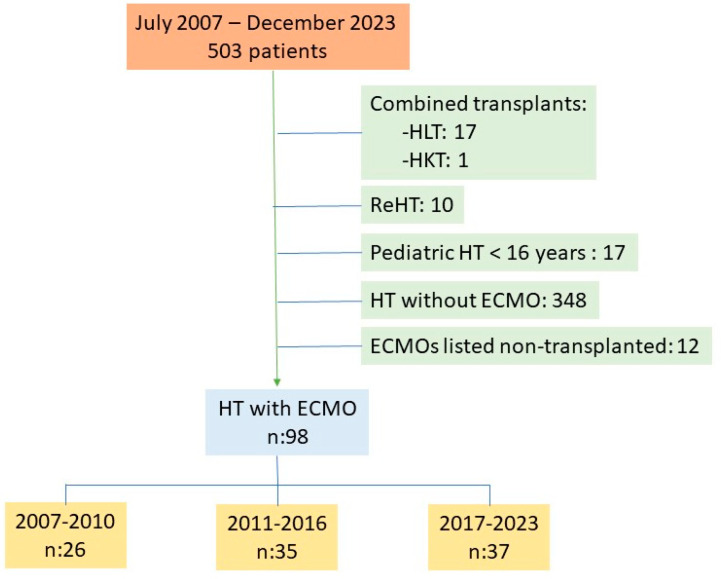
Study flowchart. Abbreviations: HLT: heart–lung transplantation; HKT: heart–kidney transplantation; and HT: heart transplantation.

This is a prospective, observational, non-interventional study in which all patients included on the heart transplant waiting list from July 2007 to December 2023 at a reference center (*n* = 503) were consecutively recruited. Patients who ultimately underwent HT without ECMO (*n* = 348), those undergoing combined HT (*n* = 18), re-transplants (*n* = 10), and pediatric transplants (<16 years, *n* = 17) were excluded. Patients who, after ECMO implantation, left the waiting list and were not transplanted, either due to death or other reasons (*n* = 12), were also excluded. Finally, 98 consecutive patients transplanted with VA-ECMO were included in the analysis. Figure 1 shows the patient flow. All transplants performed were recorded with variables considered in the Spanish Cardiac Transplant Registry [9]. Additionally, variables related to mechanical assistance were analyzed: type, days of assistance, related complications, etc. Urgency 0 or Code 0 implies priority over all other candidates nationwide to receive the first suitable available heart donor in the system and applies to patients in CS requiring short-term circulatory/ventricular assistance. The analysis was divided into time periods: the first from 2007 (the first VA-ECMO implanted as a bridge to transplant) to 2010. This period was chosen because the literature shows that the learning curve period in ECMO-implanting teams is about 3 years [10,11]. The other periods were from 2011 to 2016 and from 2017 to 2023. 

**Figure 2:**
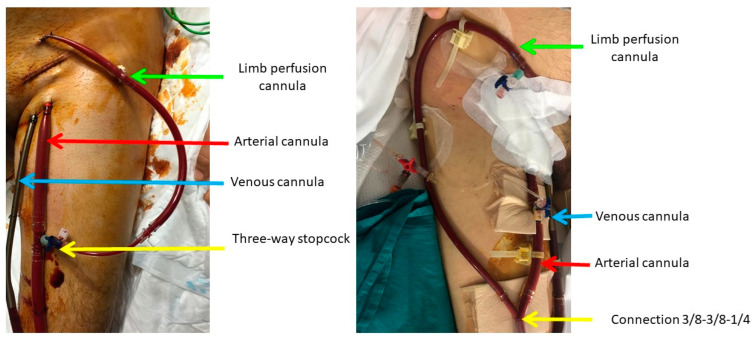
Venoarterial ECMO cannulation.

Surgical cannulation was performed in the Intensive Care Unit as per the center’s protocol, avoiding the need to transfer the unstable patient in CS to the operating room. In most patients, femoral access is used, with an arterial cannula introduced into the common femoral artery and a multi-perforated venous cannula into the femoral vein. For limb perfusion, a pediatric arterial cannula is used and connected to the ECMO arterial line. Some patients with a reduced femoral artery caliber require the placement of a Dacron graft, through which an arterial cannula is introduced, enabling simultaneous systemic and limb perfusion. In the past, the limb perfusion cannula was connected to the arterial cannula via a three-way stopcock, but now a 3/8-3/8-1/4 connection is used from the ECMO arterial line to arterial and limb perfusion cannulas to avoid the resistance that occurred with the three-way stopcock and to reduce thrombotic complications and limb ischemia. The cannulas are inserted into the femoral vessels after creating a purse-string suture with polypropylene, which is then secured with tourniquets tied to the arterial cannulas to prevent movement. In contrast, the venous cannula is not fixed to allow for repositioning if needed (Figure 2). Transthoracic echocardiography is used to confirm the correct positioning of the venous cannula entering the right atrium. Additionally, the cannulas are inserted through cutaneous counter incisions to allow for wound closure and prevent infection until ECMO weaning is possible. The procedure is performed with systemic heparinization using sodium heparin at a dose of 1 mg/kg. After 24–48 h, if no bleeding complications arise, the continuous infusion of sodium heparin is initiated.

Early mortality was defined as that occurring within the first 30 days after HT. Hospital mortality was defined as death from any cause before discharge from HT admission. Mortality from any cause was also considered at one year and five years post-transplant. Primary graft dysfunction (PGD) was defined, according to the consensus published by ISHLT in 2014, as primary graft dysfunction excluding causes such as hyperacute rejection, pulmonary hypertension, or surgical complications, diagnosed within 24 h post-surgery [12]. Severe PGD, defined as requiring post-transplant mechanical assistance, was analyzed, which, in all cases, was performed using VA-ECMO. In the study center, in urgent HT with ECMO, it is maintained by the protocol for at least 24 h post-HT; however, PGD was only considered if the previous definition was met, including the verification of ventricular dysfunction with the inability to withdraw ECMO support in the first 24–48 h post-transplant. The definitions of the complications are detailed below. Infections were considered infectious processes with or without an identified microorganism that presented with symptoms, signs, or biomarkers of infection and required the initiation or expansion of antibiotic treatment for control. All cases of pulmonary embolism (PE) were confirmed by angio-CT ± pulmonary tree angiography, while all cases of deep vein thrombosis (DVT) were confirmed by vascular Doppler ultrasound. Post-transplant renal failure (RF) was defined as that requiring renal replacement therapy (RRT), including ultrafiltration and/or dialysis, at any time during transplant admission. Vascular complications included all complications related to the vascular access of the mechanical assistance(s) used by the patient. The study protocol was approved by the Ethics Committee of Hospital Universitari i Politècnic La Fe (Code ECMO-HF), Valencia. 

### Statistical Analysis

Qualitative variables are expressed as numbers and percentages, and quantitative variables as median and interquartile ranges (non-normal distribution, *p* < 0.05 in the Kolmogorov–Smirnov test). A comparison between quantitative variables was performed using Kruskal–Wallis ANOVA. For comparative analysis between qualitative variables, Pearson’s Chi-square test was applied. Survival curves were calculated using the Kaplan–Meier method. A *p*-value of <0.05 was considered significant. Statistical analysis was performed using SPSS Statistics Version 27^®^ software and Stata Statistics/Data Analysis 16.1, serial number 501606323439. Graphs were created using SPSS and PowerPoint. The database was designed with Excel and completed at patient discharge. PowerPoint and Excel are part of the Microsoft Office Professional Plus 2019 statistical package.

## 3. Results

### 3.1. Baseline Characteristics

The profile of the patients did not show significant variations over time (Table 1). The patients’ age was similar across the three periods, around 55 years, and most were male, with ischemic heart disease being the predominant underlying etiology. However, from 2017 to 2023, the percentage of patients with ischemic heart disease was similar to those with dilated cardiomyopathy, familial, and other etiologies. The percentage of patients transplanted with mechanical ventilation (MV) was 64% and was lower in the most recent period (36% from 2017 to 2023). The duration of ECMO support was longer in the more recent periods, with a shorter duration (134 h) from 2007 to 2010. There were no differences in the main donor characteristics or surgical procedures, except for the use of the bicaval technique, which became increasingly common, reaching 89% in the last period.

### 3.2. Analysis of Overall Mortality and by Periods

**Figure 3:**
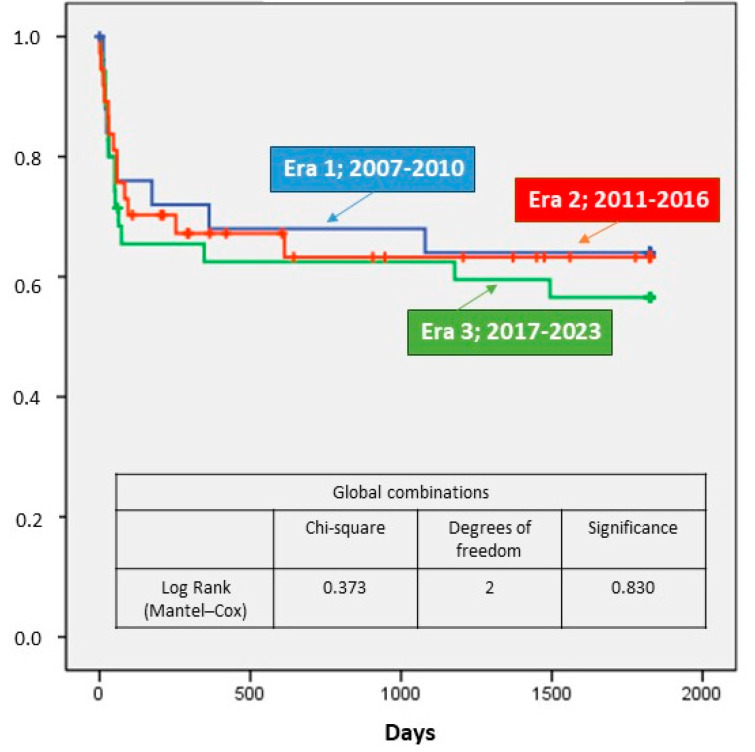
Survival at thirty days, one year, and five years.

Early mortality (day 30) did not significantly change over the years and was 19% from 2007 to 2010 and 14% from 2017 to 2023 (*p* = 0.822). No significant differences were found in mortality until hospital discharge (overall series: 25%), at one year (overall series: 36%), and five years (overall series: 39%). These results are shown in Table 2. Figure 3 shows survival over the study period for the three analyzed periods.

### 3.3. Analysis of Morbidity

**Figure 4:**
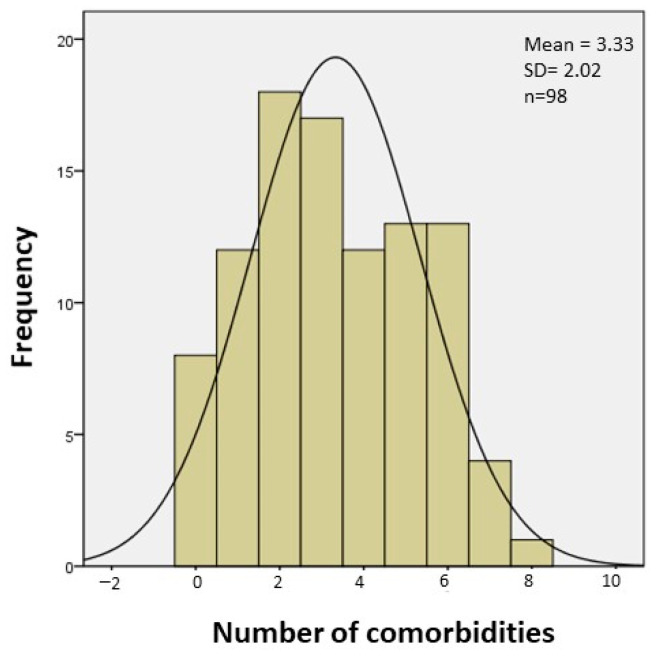
Overall prevalence of complications in patients pre-transplant (complications per patient). The histogram shows the percentage of patients presenting each number of complications.

**Figure 5:**
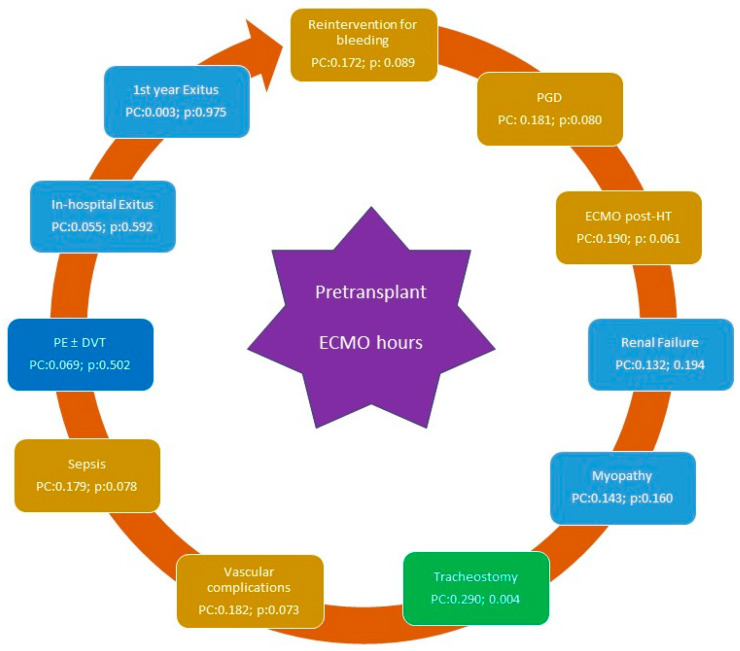
Association between complications and duration of pre-transplant ECMO support. Statistically significant associations are shown in green, trends in yellow, and associations without statistical significance in blue (Pearson correlation). Abbreviations: DVT: deep vein thrombosis; ECMO: extracorporeal membrane oxygenation; EP: pulmonary embolism; PC: Pearson correlation; PGD: primary graft dysfunction.

The overall prevalence of complications in patients with pre-transplant ECMO was generally very high, with an average frequency of 3.3 complications per patient. The percentage of patients without any of the analyzed complications was less than 10% (Figure 4). No differences were observed in the development of PE, DVT, PGD, vascular complications, or infections/sepsis (Table 3). However, in the periods from 2011 to 2016 and 2017 to 2023, there was a trend toward a lower prevalence of renal failure and a higher proportion of patients requiring tracheostomy and reoperation for bleeding and/or tamponade. In the periods from 2011 to 2016 and 2017 to 2023, there was a higher frequency of severe myopathy and the need for post-transplant mechanical support, both of which were statistically significant (*p* = 0.004 and *p* = 0.0001, respectively). The duration (hours) of pre-transplant ECMO correlated with the need for tracheostomy, with a trend toward other complications, such as the need for post-transplant ECMO, PGD, vascular complications, reoperation for bleeding, and sepsis (Figure 5).

## 4. Discussion

CS represents the most extreme form of cardiac failure, where inadequate cardiac output compromises the perfusion of tissues and organs. Short-term mortality for patients with heart failure (HF) remains above 30% [13,14,15]. In this situation, in addition to inotropic drugs, ventricular and/or circulatory assist devices are required while determining the most appropriate therapeutic approach. In fact, in these patients, especially those at INTERMACS 1 [16], the direct implantation of long-term left ventricular assist devices is associated with a high mortality rate, around 60–70% in the first year. For this reason, in these profiles, it is recommended to implant short-term mechanical support until the patient is more stabilized, either as a bridge to heart transplantation or as a bridge to long-term ventricular assist devices [17,18]. For the most urgent cases that require rapid action, ECMO has marked a turning point by reducing mortality in these patients, particularly when circulatory support is needed while awaiting the appearance of a donor as a bridge to HT [19]. However, this device, in its venoarterial variant, is not without complications and morbidity. This study aimed to assess, over a prolonged series of 17 years, the survival of patients with VA-ECMO who undergo transplantation across various eras and the comorbidities associated with the use of this device. It has been observed that, over the years, the clinical characteristics of patients have not varied, with a hospital mortality rate of 25% and an average of 3.3 relevant comorbidities per patient. 

The average clinical profile of patients in this series is a male around 55 years old. Other studies offer a slightly higher age range [20,21,22]. The majority of them reach transplantation without mechanical ventilation in the most recent period (32%). This fact reduces the risk of mortality [23,24]. In the Spanish ASIS-TC study, the average waiting time with short-term assistance until urgent CT was 7.6 days [21], which is a time similar to that of our series, which stood at 8 days, although it should be noted that 10% were excluded from the waiting list and were not transplanted. There were no differences regarding the main characteristics of the donor or the surgical procedure, except for the use of the bicaval technique. The bicaval technique in heart transplantation offers several advantages over the classical (biatrial) technique, such as the better anatomical alignment of the recipient’s atria compared with those of the donor, the preservation of the anatomy of both atria, the reduced incidence of tricuspid regurgitation due to the preservation of the integrity of the annular anatomy, and improved sinus node function [25,26]. However, the choice of technique may depend on the surgeon’s experience and the specific characteristics of the patient and donor. 

In the analyzed series, early mortality did not significantly vary over the years, being 19% from 2007 to 2010 and 14% in the most recent period. This early mortality is lower than that observed in analyses conducted on urgent HT with ECMO. In the ASIS-TC study, in-hospital mortality was 33.3% in this patient group [21]. In a study by Rousse N et al., with fewer patients, in-hospital mortality in this group was 38.5% [27]. It is very likely that early mortality in transplantation with ECMO is significantly influenced by the selection of the candidate patient and the experience of the center. Medium- and long-term mortality (1 and 5 years) is also similar across periods. A 2020 study analyzed heart transplants in adults performed in the United States between 2005 and 2017 using the UNOS database; a survival analysis was conducted to compare patients bridged to transplantation with different modalities. Of the 24,905 adult transplants performed, unadjusted 1-year post-transplant survival was 68 ± 3% in ECMO, which is similar to the overall rate in this series (64%) [28]. Lui et al. conducted a recent study using the UNOS database of all adult patients requiring VA-ECMO support before HT between 2001 and 2018; with 118 ECMO-supported transplants, a significant decrease in 1-year survival was found [29]. Overall, mortality in our series was lower than that reported in other studies. In this regard, it should be noted that a recent study found higher mortality rates in centers that did not perform long-term transplantation/assistance (65.5% compared to 55.8% in centers that performed long-term transplantation/assistance) [30]. It should be noted that it is known that urgent heart transplants have a worse initial prognosis, while in the long term, patients who survive tend to have a better prognosis, as they are usually younger and more carefully selected patients. In the latest report from the Spanish Heart Transplant Registry, urgent HT increases the mortality of the procedure with a hazard ratio of 1.3 [31].

The overall prevalence of complications in patients with pre-transplant ECMO was generally high, with an average frequency of 3.3 complications per patient. There were no observed differences between eras in the development of PE, DVT, acute limb ischemia, femoral arteriovenous fistula, femoral stenosis, vascular complications, or infections/sepsis. A recent study evaluated the frequency of vascular complications (arterial and venous) following ECMO removal using Doppler ultrasound on all patients, with a median support duration of 8 days; DVT was found in 41% and arterial complications in 14% (including 9 cases of acute limb ischemia, 1 femoral arteriovenous fistula, and 5 cases of late femoral stenosis) [32]. This series collectively analyzed DVT and vascular complications, finding a prevalence of 54% in the last era analyzed. Regarding post-transplant infections in patients undergoing urgent HT, it is estimated that infectious complications could affect slightly more than half of the ECMO-treated patients and those using short-term mechanical assist devices [33,34]. In this series, the prevalence of post-transplant infections in urgent ECMO cases was 29%, which is similar to that reported in a recent multicenter study, which found that nearly 35% of patients undergoing urgent CT with short-term assistance had a total of 102 infections, 26% of which involved ECMO [35]. Infections are estimated to complicate between 30% and 55% of ECMO treatments and impact survival [34,36]. Ventilator-associated pneumonia is the most frequent nosocomial infection in patients receiving veno-venous ECMO [36]. The lack of a standardized definition for ECMO-related infections, differentiation between colonization and infection, and unreliable clinical markers of infection during ECMO make understanding the relationship between infection and outcomes a challenging and not fully clarified task [37]. Regarding the need for post-transplant ECMO, it was higher after 2011 (over 70%), but it is important to note that, in our center, post-transplant ECMO is protocol-mandated for at least 24 h in patients transplanted urgently with ECMO support. This approach is based on the fact that urgent HT with ECMO is associated with higher rates of PGD [38]. Therefore, maintaining ECMO could help preserve hemodynamics and stability in patients during the first few hours post-transplant. As described in the Methodology section, sustained mechanical support was not considered PGD but rather confirmed ventricular dysfunction with an inability to remove ECMO support within the first 24 h post-transplant. PGD occurred in 19% of the entire series, rising to 24% in the latest era. This prevalence is lower than that reported in studies conducted with ECMO in our setting, where prevalence exceeded 30% [21]. PGD is a severe complication and the leading cause of death within the first 30 days after transplantation [31]. It is known that PGD is much more frequent in urgent HT compared to elective cases, and within urgent HT, it is more common in patients arriving for HT with mechanical assistance [39,40]. The likely explanation for the relationship between ECMO assistance and PGD is linked to the systemic effects of ECMO. ECMO support is associated with immunological alterations (increased circulating immature neutrophils, lymphocyte dysfunction, etc.) and leads to elevated levels of pro-inflammatory cytokines, such as interleukins and tumor necrosis factor-alpha [41]. There is a trend observed in the periods from 2011 to 2016 and 2017 to 2023 towards a lower prevalence of renal failure and a higher proportion of patients requiring tracheostomy and reoperation due to bleeding and/or tamponade. The exact reasons for the reduction in renal failure over the years are complex and heterogeneous, involving pre-existing renal disease, acute injury during surgery, and calcineurin inhibitor toxicity. Routine ECMO maintenance in the early hours may have favored renal perfusion and minimized the deleterious effects of surgery and the onset of immunosuppression. The increased need for tracheostomy in recent eras may be influenced by myopathy, the waiting times until an organ is obtained, and pre-transplant mechanical ventilation, as mentioned earlier. Regarding reoperations for bleeding, it should be noted that the exposure of blood to non-biological components of the circuit activates the coagulation system and degrades hemostatic factors [42]. This, coupled with systemic anticoagulation requirements, potential thrombocytopenia, hypofibrinogenemia, and the shear-mediated loss of key platelet surface molecules like selectin and high molecular weight von Willebrand multimers [43], contributes to these complications. Lastly, concerning complications, there is a higher frequency of severe myopathy observed in the periods from 2011 to 2016 and 2017 to 2023, likely due to increased ECMO duration over the years until organ procurement and the impact of mechanical ventilation. Mechanical ventilation, besides being associated with post-transplant morbidity and mortality, reflects a pre-transplant state of myopathy that limits spontaneous ventilation. There is extensive information in the literature regarding complications associated with long-term assist devices from clinical trials and multicenter registries. However, fewer studies have systematically addressed the incidence and clinical impact of complications associated with short-term mechanical circulatory support devices. The high incidence of complications is probably the main drawback of short-term assist devices, and there is evidence that these complications have a significant impact on post-transplant mortality. 

Following the analysis of survival and complications, it can be stated that urgent HT with ECMO is a tool that has undoubtedly changed the prognosis of severe HF and urgent HT bridging, offering hopeful survival outcomes for these critically ill patients, albeit with considerable morbidity. The establishment of multidisciplinary teams (ECMO Teams) has allowed for the standardization of processes and appropriate patient selection in this complex scenario [44]. This study has several limitations. Firstly, the number of patients included in the analysis is not high, and they are all from a single center. Secondly, patients with ECMO listed for transplantation who were excluded before the procedure due to multiorgan failure were not considered. Had these patients been transplanted, their mortality and morbidity might have been increased due to their exclusion at the point where transplantation was deemed unfeasible because of multiorgan failure, leading to their subsequent demise. However, the single-center nature of this study ensured a homogeneous and consistent protocol for all patients, likely explaining the absence of differences when comparing different eras. Additionally, it is a series with a sufficient number of cases from a center with extensive experience in cardiac transplantation and ECMO implantation. The prospective nature of this study also lends reliability to the results.

## 5. Conclusions

VA-ECMO support as a bridge to HT allows discharge from the hospital for three out of every four transplanted patients. This survival rate has remained unchanged over the years. On the other hand, it should be noted that the comorbidities associated with this device are frequent and significant.

## Figures and Tables

**Table 1:** Clinical characteristics of patients (overall and by eras).

	2007–2010 (n: 26)	2011–2016 (n: 35)	2017–2023 (n: 37)	*p*	Overall (n: 98)
**Recipient**					
Age (years) #	58 (11)	54 (21)	51 (14)	0.242	55 (15)
Male (n, %)	16 (62)	26 (74)	28 (76)	0.425	70 (71)
Underlying etiology (n, %)				0.474	
ischemic	13 (50)	18 (51)	13 (35)		44 (45)
Idiopathic + familial DCM	7 (27)	11 (31)	13 (35)		31 (32)
Other	6 (23)	6 (18)	11 (30)		23 (23)
Creatinine (mg/dL) #	0.9 (0.8)	0.7 (0.7)	0.7 (0.3)	0.124	0.8 (0.6)
AST (U/L) #	70 (53)	55 (36)	39 (39)	0.178	54 (45)
ALT (U/L) #	59 (94)	38 (44)	47 (83)	0.365	51 (69)
Bilirrubin (mg/dL) #	1.4 (1.5)	2.2 (1.9)	1.3 (1.5)	0.004	1.6 (1.6)
Pre-transplant infection (n, %)	8 (31)	13 (37)	7 (19)	0.254	28 (29)
I-D Diabetes (n, %)	0 (0)	3 (9)	2 (5)	0.445	5 (5)
MV (n, %)	26 (100)	25 (71)	12 (32)	0.001	63 (64)
Previous sternotomy (n, %)	2 (7)	5 (14)	6 (16)	0.724	13 (13)
ECMO duration (hours) #	134 (128)	204 (219)	192 (120)	0.004	192 (147)
**Donor**					
Donor age (years)	43 (24)	46 (16)	46 (15)	0.173	45 (17)
Donor cause of death (n, %)				0.345	
TBI	5 (19)	10 (29)	9 (24)		25 (24)
Stroke	20 (77)	23 (66)	22 (59)		65 (66)
Other	1 (4)	2 (5)	6 (17)		9(10)
**Surgical Procedure**					
CPB time (min) #	135 (53)	139 (42)	115 (39)	0.107	128 (43)
Ischemia time (min) #	200 (50)	192 (97)	189 (79)	0.444	192 (83)
Bicaval technique (n, %)	14 (54)	24 (69)	33 (89)	0.002	71 (72)

# Median and interquartile range. Statistical test: Kruskal–Wallis ANOVA and Pearson’s Chi-square test. Abbreviations: ALT: alanine aminotransferase; AST: aspartate aminotransferase; CPB: cardiopulmonary bypass; DCM: dilated cardiomyopathy; ECMO: extracorporeal membrane oxygenation; I-D: insulin-dependent; MV: mechanical ventilation; TBI: traumatic brain injury.

**Table 2:** Mortality analysis.

Variables	2007–2010 (n: 26)	2011–2016 (n: 35)	2017–2023 (n: 37)	*p*	Overall (n: 98)
Mortality at 30 days (n, %)	5 (19)	6 (17)	5 (14)	0.822	16 (16)
In-hospital mortality (n, %)	6 (23)	9 (26)	9 (24)	0.972	24 (25)
Mortality at one year (n, %)	9 (35)	13 (38)	12 (36)	0.706	34 (36)
Mortality at five years (n, %)	10 (39)	15 (43)	13 (35)	0.797	38 (39)

In each period, the mortality values are cumulative. Statistical test: Pearson’s Chi-square test.

**Table 3:** Morbidity analysis.

Variables	2007–2010 (n: 26)	2011–2016 (n: 35)	2017–2023 (n: 37)	*p*	Overall (n: 98)
Renal failure (n, %)	15 (58)	12 (34)	15 (41)	0.177	42 (43)
DVP ± EP (n, %)	2 (8)	0 (0)	4 (11)	0.058	6 (6)
PGD (n, %)	5 (19)	5 (14)	9 (24)	0.560	19 (19)
Tracheostomy (n, %)	4 (15)	11 (31)	13 (35)	0.208	28 (29)
Severe myopathy/polyneuropathy (n, %)	8 (31)	19 (54)	27 (73)	0.004	54 (55)
Reintervention for bleeding/tamponade (n, %)	6 (23)	17 (49)	18 (49)	0.077	41 (42)
Post-transplant ECMO (n, %)	5 (19)	25 (71)	26 (70)	0.001	56 (57)
Vascular complications * (n, %)	16 (62)	21 (60)	20 (54)	0.808	57 (58)
Sepsis/infection (n, %)	6 (23)	12 (34)	10 (27)	0.610	28 (29)

* Includes hemorrhages and compartment syndrome. Statistical test: Pearson’s Chi-square test. Abbreviations: DVT: deep vein thrombosis; ECMO: extracorporeal membrane oxygenation; PE: pulmonary embolism; PGD: primary graft dysfunction.

## Data Availability

The dataset is available upon request to the authors.
